# Non-allopathic adjuvant management of osteoarthritis by alkalinisation of the diet

**DOI:** 10.4102/phcfm.v7i1.780

**Published:** 2015-04-21

**Authors:** David P. van Velden, Helmuth Reuter, Martin Kidd, F. Otto Müller

**Affiliations:** 1 Department of Pathology, Faculty of Medicine and Health Sciences, Department of Pathology, Stellenbosch University, South Africa; 2 Winelands Medical Research Centre, South Africa; 3 Centre for Statistical Consultation, Department of Statistics and Actuarial Sciences, Stellenbosch University, South Africa; 4 Clinical Trials and Drug Development Consultant, George, South Africa

## Abstract

**Background:**

Osteoarthritis (OA) is a chronic condition. Nonsteroidal anti-inflammatory drugs recommended for treatment have serious adverse effects. A compelling body of anecdotal evidence alerted the authors to the therapeutic potential of dietary supplementation with Multiforce^®^ (MF) Alkaline Powder for relief of OA symptoms.

**Aim:**

The aim of the study was to test the hypothesis that dietary supplementation with MF relieves clinical signs and symptoms of OA of the hands.

**Setting:**

The study was done at the MEDSAC hospital in Somerset West, Western Cape, South Africa.

**Methods:**

The research was conducted in two stages. An open interventional study (*n* = 40) confirmed the notion that MF 7.5 g twice daily is likely to be an effective alternative or adjunct for relief of symptoms of OA of the hands. The main study was conducted with 100 eligible, consenting volunteers (aged 47–89 years) according to a randomised, placebo-controlled, crossover design. Study duration was 56 days, 28 days per regimen; crossover to alternate regimens took place on day 28.

**Results:**

Compared to placebo, MF intake over 28 days was associated with significant reductions (*p* < 0.005) in pain, tenderness and stiffness of interphalangeal and metacarpophalangeal joints of the hand. Confirmation of systemic alkalinisation by MF, which is rich in organic anions in the form of citrate salts, was reflected by a significant and sustained increase in urine pH.

**Conclusion:**

A dietary supplement, Multiforce^®^ Alkaline Powder, containing citrate salts which are converted into bicarbonate *in vivo*, was efficacious and safe as sole therapeutic intervention, significantly attenuating OA-associated signs and symptoms of the hands.

## Abstrait

**Gestion adjuvante non-allopathique de l’ostéoarthrite par alcalinisation de l’alimentation.**

**Introduction:**

L’ostéoarthrite (OA) est une condition chronique. Les médicaments anti-inflammatoires non-stéroïdes – piliers du soulagement symptomatique – peuvent causer des effets nocifs graves, surtout chez les personnes âgées. Un ensemble de preuves anecdotiques probantes ont attiré l’attention des auteurs sur le potentiel thérapeutique du supplément alimentaire du Multiforce^®^ Alkaline Powder (MF) pour le soulagement des symptômes d’ostéoarthrite.

**Objectifs:**

Le but de l’étude était de tester, au moyen d’essais contrôlés randomisés, l’hypothèse que le supplément alimentaire avec MF soulage les signes et les symptômes cliniques de l’OA des mains.

**Méthodes:**

La recherché a été faite en deux phases. Une étude ouverte interventionnelle (*n* = 40) a confirmé la notion que 7.5 g de MF deux fois par jour est une alternative ou un supplément efficace pour le soulagement des symptômes d’ostéoarthrite des mains. Là-dessus l’étude principale a été faite sur 100 volontaires admissibles et consentants (47−89 ans), selon un plan croisé, randomisé et contrôlé par placebo; *n* = 50 par groupe de traitement A et B (verum *n* = 50- groupe A, et placebo *n* = 50- groupe B, spécifiant quel traitement a été fait en premier). La durée de l’étude était de 56 jours, 28 jours par régime. La transition aux régimes alternés s’est faite le 28^ème^ jour. Les visites cliniques ont eu lieu tous les quinze jours.

**Résultats:**

Comparativement au placebo, la prise de MF pendant 28 jours a montré des réductions significatives des douleurs (*p* < 0.005), de la sensibilité et de la raideur des articulations inter-phalangiennes et métacarpo-phalangiennes de la main. La confirmation d’une alcalinisation par MF, riche en anions organiques sous forme de sels de citrate, a été vérifiée par une augmentation importante et prolongée du pH de l’urine. La prise de MF a été bien tolérée.

**Conclusion:**

Le supplément alimentaire, Multiforce^®^ Alkaline Powder, contenant des sels de citrate qui sont convertis en bicarbonate in vivo, est efficace et sans danger quand il sert de seule thérapie, et il atténue considérablement les signes et les symptômes associés à l’ostéoarthrite des mains.

## Introduction

Osteo-arthritis (OA) is a major cause of morbidity, affecting 60% of men and 70% of women over the age of 65 years.^1^ Current therapeutic approaches, including allopathic (Western) medicine, fail to prevent initiation and progression of OA, and some have life-threatening side-effects. The rapidly rising rates of complex, chronic disease are creating an unsustainable burden on the national economy in both direct (e.g. treatment) and indirect (e.g. lost productivity) costs.

These chronic and degenerative diseases cannot be effectively treated with drugs alone. As a matter of fact, chronic drug use for these conditions is not only costly but often has serious side-effects. Popular analgesics and nonsteroidal anti-inflammatory drugs (NSAIDs) such as celecoxib, diclofenac, ibobrufen, naproxen and aspirin have confirmed risk for heart disease and internal bleeding.^2^,^3^ Paracetamol can damage the liver. Allopathic medicines often do not cure these diseases, but rather mask the symptoms.

The incidence of OA increases with age, and ageing patients present with comorbidities that add to the complexity of treatment. Degeneration of joint cartilage is still the most important pathophysiological feature of OA. Tissues surrounding the joints, such as muscles, bones, tendons and ligaments, are also involved in the disease process. Recommendations for management of OA comprise non-pharmacological and pharmacological approaches. Non-pharmacological interventions include education and self-management, referral to a physical therapist, aerobic muscle-strengthening and water-based exercises, weight reduction, diet interventions, walking aids, knee braces, therapeutic footwear and insoles, thermal modalities, transcutaneous electrical nerve stimulation and acupuncture. Pharmacological treatments consist of paracetamol, systemic and topical cyclo-oxygenase-2 (COX-2) non-selective and selective inhibitors, classified as NSAIDs, topical capsaicin, intra-articular corticosteroids and hyaluronates, glucosamine and/or chondroitin sulphate and diacerin for possible structure-modifying effects, and use of opioid analgesics and tramadol for the treatment of refractory pain.^4^,^5^ Methotrexate, which has immunosuppressive and anti-inflammatory effects, is promising, but this drug may have serious side-effects, including liver disease, lung inflammation, increased susceptibility to infection, and suppression of blood cell production in the bone marrow. Studies with disease-modifying drugs in management of OA, such as bisphosphonates with the aim of inhibiting increased bone turnover, did not produce positive results.^1^

Long-term use of systemic NSAIDs to relieve OA symptoms could cause serious adverse events, such as gastro-intestinal bleeding, renal damage, and induction or aggravation of bronchial asthma and cardiovascular complications.^2^ The effect of COX-2 inhibitors on renal function is still tenuous. Of great concern has been the voluntary withdrawal of rofecoxib (Vioxx^®^) from the market in 2004 because of an increased risk of serious cardiovascular events, including heart attacks and stroke amongst patients taking Vioxx^®^ compared to patients receiving placebo.^6^

### Dietary considerations

The typical ‘Western diet’ is considered acidogenic due to the greater acid load contained in animal products, and is low in fruit and vegetables, resulting in a state of overlooked low-grade chronic, compensated metabolic acidosis.^7^ The ensuing acidotic stress and hypoxia may play a role in the pathophysiology of OA.^8^ In response to states of diet-derived metabolic acidosis, the kidney implements compensating mechanisms to restore the acid-base balance.

In South Africa there is compelling anecdotal evidence that an alkaline diet supplement, Multiforce^®^ (MF) Alkaline Powder, has beneficial effects in patients with primary OA prompted this research to objectively explore the influence of dietary supplementation with MF on symptoms and signs of OA.

### Aim

The aim of the study was to test, by means of a randomised controlled trial, the hypothesis that dietary supplementation with MF relieves clinical signs and symptoms of OA of the hands.

## Research methods and design

The research comprised two stages, a pilot study and the main study.

In the pilot, a single-centre, open study of the efficacy and safety of MF in participants (*n* = 40) with OA revealed significant improvements (*p* < 0.005) in all parameters assessed. Side-effects were negligible (unpublished data).

The pilot study was followed by a single-centre, double-blind, placebo-controlled crossover study of the efficacy and safety of MF in participants with OA of the hands (*n* = 100), which is the subject of this article.

Both protocols were approved by the Ethics Committee for Human Research of Stellenbosch University (SU Ethics ref no: 10/02/009).

### Study population

One hundred (100) consenting and responsible adult male and female volunteers (47–89 years of age; mean 65 years) with symptoms and signs of OA of the hands (left or right), with or without current allopathic treatment, fulfilling the inclusion criteria were recruited in Somerset West and surrounding areas.

For inclusion participants had to be able to understand and follow the instructions of the protocol; be mobile enough to attend visits to the clinic; give informed consent; be willing, committed, and able to return for all clinic visits and complete all study-related procedures; be able to engage in telephone communication; be compliant with the American Rheumatism Association classification of OA;^9^ and be able to complete a daily pain visual analogue scale (VAS) and the validated Stanford Health Assessment Questionnaire (HAQ) 20-item disability scale at each of the six visits. The two-page HAQ-DI (disability index)^10^ was used.

Exclusion criteria were, inter alia, gout or serum uric acid equal to or greater than 0.45 mmoL/L, rheumatoid arthritis or high-sensitivity C-reactive protein (hs-CRP) > 20 mg/dl, known or suspected current infection or recurrent infectious disease of a joint, immunosuppressive therapy, history of demyelinating disease or symptoms suggestive of multiple sclerosis, psoriasis, intra-articular, intramuscular or intravenous glucocorticoid therapy eight weeks prior to the screening visit, glomerular filtration rate of < 30 mL/min (as per the Modification of Diet in Renal Disease index), history of acute or recurrent urinary tract infections, ‘abnormal’ clinical chemistry and haematology values, such as serum transaminases and alkaline phosphatase > 2 x upper limit of normal, full blood count outside normal limits, and abnormal serum electrolyte levels.

In addition, patients with any other arthritic or medical condition that in the opinion of the investigator could compromise participation or interfere with evaluations were excluded, as were those with a history of drug abuse within the five years prior to the screening visit, a history of alcohol abuse or current intake of 21 or more alcohol-containing drinks per week, and any investigational drug taken within three months or five drug half-lives, whichever was the longer period, prior to screening.

### Washout period

Prior to randomisation eligible participants were instructed to discontinue any medication (over-the-counter or prescription) used for relief of pain or stiffness of joints for 14 consecutive days, and preferably with the consent of a prescribing physician. At completion of the study patients could be switched back to their pre-study medication regimen.

### Screening visit (visit 1)

At the screening visit participants provided informed consent and underwent clinical and laboratory evaluation for trial inclusion. In addition a urine dipstick analysis was performed and urine pH measured by means of a semi-automated colorimetric method (Siemens kit) within two hours of voiding. Patients were instructed not to change their diet in any way during the study period. Random urinary pH was an indication of the dietary acid load; 85% of study participants had a low urinary pH, indicating an acidogenic diet.^11^

### Trial medication

Study-related medication (MF verum and matching placebo) was supplied by Bioforce SA Pty (Ltd). Each 7.5 g of MF Alkaline Powder (verum) contains magnesium hydrogenium phosphate 244 mg, calcium citrate 145 mg, potassium bicarbonate 783 mg, magnesium citrate 315 mg, potassium citrate 870 mg, dicalciumphosphate 2-hydrate 973 mg, organic plant calcium, acerola extract and mannitol, and delivers about 250 mg of elemental calcium. The placebo contained mannitol, polydextrose, pirosil, xanthan gum, maize starch and beetroot leaf mix, and was designed and manufactured by Powdermix Technologies (Pty) Ltd (www.powdermix.co.za).

Trial medication was provided in sachets containing 7.5 g of product. The contents of one sachet, suspended in 200 mL of non-carbonated water at room temperature, were ingested twice daily; that is 30 minutes before breakfast (am) and 30 minutes before supper (pm). Patients were instructed to take the medication (verum or placebo) for a period of 28 days, after which they immediately switched to the alternative treatment for another 28 days. The participants and evaluating physician were blinded regarding the identity of treatments.

### Dietary intervention

Patients were encouraged to continue with their individual standard diets for the duration of the study, the only intervention being supplementation with MF.

### Biochemical assessment of inflammation

At each visit hs-CRP measurement was performed to evaluate whether subjects with long-established OA had active inflammation, and to assess whether MF intervention had any anti-inflammatory effects.^12^

### Assessment of joints

At the end of the washout period assessment of generalised pain was done according to a VAS. Pain assessment was done on a 10-point scale, which is a double-anchored horizontal line standardised to 15 centimetres in length, where each end represents opposite ends of a continuum. This line is labelled 0 = no pain at the left anchor point and 10 = severe pain at the right anchor point. Patients were instructed to place a vertical mark on the line to indicate the severity of their pain.

The interphalangeal or metacarpophalangeal joints of the hand were evaluated separately. One joint only served as outcome target; that is left or right hand (interphalangeal or metacarpophalangeal joints), whichever was the worst affected. The affected joints were assessed for tenderness on pressure, and for pain and stiffness upon active or passive movement. Tenderness, pain and stiffness were recorded on a 4-point scale as follows:

- 0 = Patient felt no tenderness/pain/stiffness.
- 1 = Patient complained of pain/stiffness.
- 2 = Patient complained of pain and winced.
- 3 = Patient complained of pain, winced and withdrew the hand.

Clinical assessments were performed by the same investigator throughout the study.

### Rescue medication

Participants were allowed to use oral paracetamol up to a maximum of 2 g daily for pain during the washout and trial periods. In the event that the intake of maximum daily quantities was exceeded on five consecutive days, patients were to be withdrawn from the study as ‘treatment failures’. No other analgesics or NSAIDs were allowed during the washout and trial periods. Reconciliation of rescue medication issued and used took place at visits 4, 5 and 6, and at any time of termination of participation.

### Concomitant medication

Participants were allowed to continue with existing chronic medication if there was no suspected interaction between MF and such regimens.

### Study flow sequence

#### Day 1 (visit 2)

Baseline clinical and biochemical data, including urine pH, were collected. Instruction was given regarding quality of life and VAS scale scoring. Randomisation was done to either MF verum or MF placebo (*n* = 50 per treatment group).

#### Day 14 (visit 3)

QOL and pain-scale data were collected. Joint parameters were clinically assessed. Urine pH was determined. Side-effects were recorded. Rescue medication was reconciled and reissued.

#### Day 28 (visit 4)

Clinical and biochemical evaluation was done as on day 1 (visit 2). Day 14 (visit 3) procedures were repeated with a crossover to the alternate regimen (verum ⇄ placebo).

#### Day 42 (visit 5)

Evaluations were done as on day 14 (visit 3).

#### Day 56 (visit 6)

This visit signalled the end of the study. Evaluations were done as per day 28 (visit 4). Patients who chose to do so switched back to pre-study pharmacotherapy.

### Statistical analysis

For statistical analysis of the data a mixed-model repeated measures analysis of variance was conducted, with group and time as fixed effects and the patients as random effects. For post-hoc pairwise comparisons, Fisher's least significant difference (LSD) was used. Analyses were conducted using the VEPAC module of Statistica 12 data analysis software system (StatSoft, Inc., 2014; www.statsoft.com). A significance level of 5% (*p* < 0.05) was used as the guideline for determining significant changes.

## Results

One hundred and fifty-nine patients were screened for OA of the hands, and 100 who fulfilled the inclusion criteria were enrolled in the study. They were randomly assigned into two groups, A and B (verum *n* = 50 group A, and placebo *n* = 50 group B, specifying which treatment came first). Drop-outs were not replaced. Two patients, one from each treatment group, dropped out because they relocated, and 98 patients completed the trial. Eighty-eight per cent of participants were female.

All participants had primary OA of the IP or MP joints of the hands.

### Acceptability and tolerance of supplement

No serious adverse events occurred. Three patients reported mild diarrhoea whilst on the verum intervention. Overall acceptability was good.

### Value of the Stanford HAQ

During the two-month intervention trial no significant changes were found in functional ability in dressing and grooming, arising, eating, walking, hygiene and reach. Most participants had only difficulty in grip strength (opening jars and taps) and in performing certain household activities and gardening. Although they did experience some improvement in the function of the hands on the verum, this was not statistically significant. It is clear that this questionnaire is more appropriate for patients with rheumatoid arthritis with severe functional disability, and it did not help in assessing the influence of the intervention on OA in those with longstanding OA and no other serious functional disabilities. Patients with OA of the hands have low functional disability not amenable to change.

### Efficacy

**FIGURE 1:**
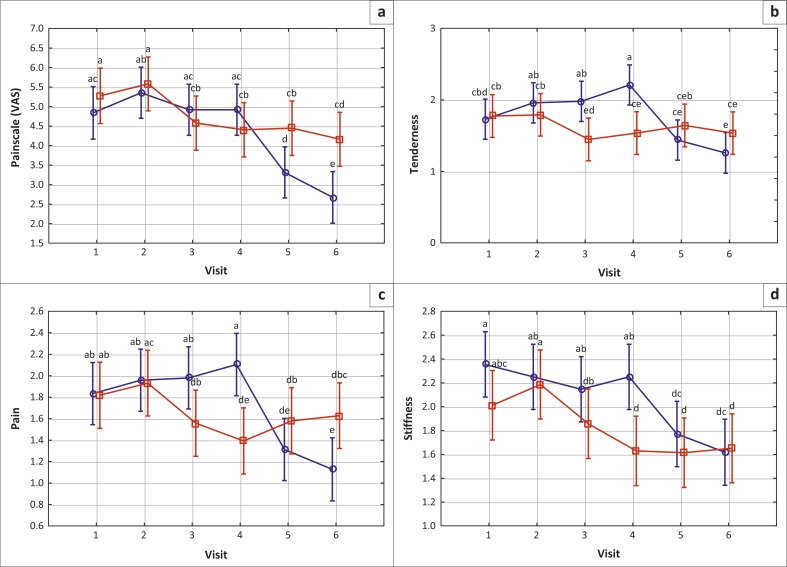
VAS pain score on 10-point scale, and clinical assessment of interphalangeal or metacarpophalangeal joint tenderness, pain, and stiffness on a 4-point scale (0–3). Vertical bars denote 0.95 confidence intervals. Blue: placebo followed by verum; red: verum followed by placebo. The letters on Figures 1 and 2 represent the results from the post hoc tests where all means were compared pairwise to determine possible significant differences. In this way any mean on the graph can be compared to any other mean. If the annotations share just one letter (e.g. a vs. a, b vs. b or a vs. ab), then the corresponding *p*-value comparing the two means will be > 0.05. If the annotations share no letters (e.g. a vs b or a vs bc) then the corresponding *p*-value comparing the two means will be < 0.05.

The influence of the verum (MF) intervention compared to placebo on efficacy parameters is depicted in Figure 1 (a–d).

The MF verum regimen, prior to or following the placebo regimen, resulted in significant (*p* < 0.005) improvement in the four efficacy variables evaluated. Significance was reached within two weeks of treatment. Placebo did not exhibit any positive outcome (*p* > 0.005) at any time. Of note is the observation that switching from verum to placebo was not accompanied by worsening of the signs and symptoms of OA, and that verum treatment had apparently not reached optimum therapeutic efficacy after four weeks.

### Urine pH

The group that received placebo first followed by a switch to verum after four weeks showed no significant change in urine pH, but the switch made after four weeks (visit 4) was followed by a significant and sustained increase in pH.

**FIGURE 2:**
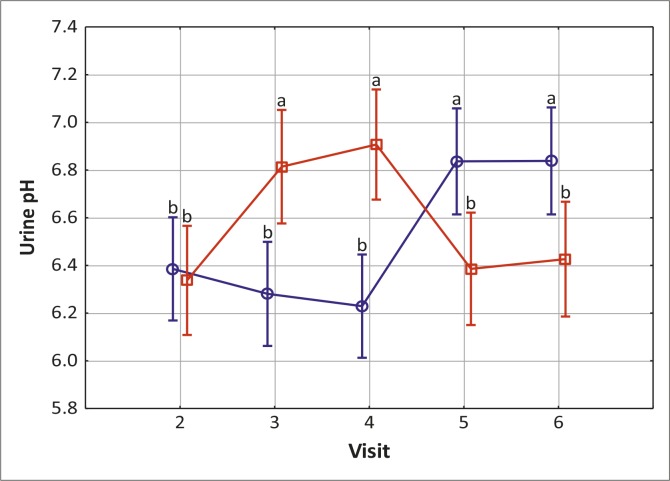
Mean urine pH values determined at baseline and subsequent fortnightly clinic visits. Blue: placebo followed by verum; red: verum followed by placebo.

Conversely, in the group that received verum first, followed by placebo after four weeks, urine pH increased significantly, but when the switch to placebo was made (visit 4) urine pH returned to pre-verum values. These data clearly distinguished verum from placebo and served as a surrogate endpoint for gauging study compliance with regard to adherence to treatment regimens (see Figure 2).

### Inflammatory marker

There was no change in hs-CRP during the study period of two months. The median value for hs-CRP at baseline was 2.9 mg/dL (25th – 75th percentile 0.3–23). Traditionally CRP has been used to distinguish systemic inflammatory disorders such as rheumatoid arthritis from OA. However, multiple studies have demonstrated that hs-CRP is modestly elevated in the plasma of patients with OA compared to age-matched controls. Whilst hs-CRP levels in rheumatoid arthritis are typically > 15 mg/L, levels in OA typically range from 3 to 8 mg/L.^12^ This may indicate that the preparation does not influence inflammation, or it may be that the volume of the synovium in longstanding OA is too small to influence it.

### Rescue medication

We investigated the use of the rescue medication and it was found not to be different in the placebo and verum groups.

## Discussion

The results of this randomised placebo-controlled study involving 98 participants corroborate the findings of the pilot study, namely that the dietary supplement MF, at a dose of 7.5 g twice daily, reduces signs and symptoms of OA in hands significantly, and that ingestion of MF in this manner is associated with alkalinisation of urine. It is thus a tenable deduction that the pilot study was devoid of a placebo effect.

Efficacy of MF reached significance within two weeks and was sustained over the four weeks of treatment, regardless of the order of randomisation. It is also apparent that efficacy levels had not reached steady state after four weeks, which suggests that MF could provide even more pronounced relief if used continuously. This theory is supported by the observation that MF treatment appeared to result in a ‘carry-over’ effect, deduced from the finding that improvement in signs and symptoms of OA was sustained even after crossover from verum to placebo.

A limitation of the study is the short intervention time, and that we did not do a diet recall during the study. Dietary restrictions and modifications were not enforced in this study. Therefore it is reasonable to deduce that alterations in the OA signs and symptoms and urine pH were induced by dietary supplementation with MF, which is rich in organic anions, notably citrate salts, which are converted into bicarbonate systemically. Although the mechanism(s) whereby MF supplementation provided clinical relief of OA of the hands is not clear, the acidic extracellular environment may lead to increased intracellular acid loads experienced by chondrocytes. Extracellular factors such as acidosis may affect articular chondrocytes and this may have implications for disease progression and potential therapeutic intervention. Articular cartilage is a highly specialised tissue designed to allow pain-free, friction-less movement across joints. Mature cartilage is avascular, relatively hypoxic and acidic and thus provides an unusual and challenging environment to the resident cell, the chondrocyte.

In joint diseases such as OA and rheumatoid arthritis oxygen levels are reduced further from increased consumption by inflammatory cells, and reduced delivery of oxygen to synovial fluid due to joint capsule fibrosis and subchondral bone sclerosis. Alteration in the physical environment and release of inflammatory mediators by articular cells (under disease conditions) leads to acidosis. Cartilage matrix synthesis has a bimodal response to alterations in extracellular pH with optimal synthesis occurring between pH 7.0–7.2, and synovial fluid acidosis occurs in OA and rheumatoid arthritis, suggesting that pH and hypoxia may have an important role in maintaining cartilage integrity.^7^ It is possible that ‘correction’ of underlying systemic metabolic acidosis resulting from ‘Western-style’ eating habits may play a role.^6^,^13^,^14^

## Conclusion

Dietary supplementation with MF, which contains organic anions in the form of citrate salts, significantly relieved the symptoms and signs of OA of the hands. Based on the study outcome, MF may be considered a safe and effective, non-allopathic therapeutic modality, alone or as an adjunct to other interventions, in the management of this common degenerative disease. Further research based on comorbidity factors might be warranted. These are short-term findings, and long-term studies are required to evaluate the influence of MF on bone and joint physiology and pathology.
